# Icariin alleviates osteoarthritis by regulating autophagy of chondrocytes by mediating PI3K/AKT/mTOR signaling

**DOI:** 10.1080/21655979.2021.1943602

**Published:** 2021-06-24

**Authors:** Yanghua Tang, Yongfu Li, Dawei Xin, Lin Chen, Zhenfei Xiong, Xuezi Yu

**Affiliations:** a Department of Orthopedics, Hospital of Traditional Chinese Medicine of Xiaoshan District, Hangzhou, Zhejiang, China; b Department of Orthopedics, The Second People’s Hospital of Tonglu, Hangzhou, Zhejiang, China; c Department of Orthopedics, Xixi Hospital of Hangzhou, Hangzhou, Zhejiang, China

**Keywords:** Icariin, osteoarthritis, rapamycin, autophagy, chondrocytes

## Abstract

Osteoarthritis (OA) is a chronic degenerative disease that significantly impacts the quality of life of the elderly population. Recently, the pathogenesis of OA has been reported to involve autophagy in chondrocytes. Intriguingly, icariin, one of the main components of epimedium, exerts multiple pharmacological effects, including a protective effect against chondrocyte damage. Thus, we aimed to investigate the therapeutic effect of icariin on OA and its potential underlying mechanism by using a rat model of OA. After treatment with icariin or an autophagy activator (rapamycin) or inhibitor (3-methyladenine), OA chondrocyte viability was measured using the CCK-8 assay, apoptosis in the chondrocytes was evaluated using the acridine orange-propidium iodide assay and flow cytometry, and OA tissue pathological state was assessed using micro-CT scanning and safranin O staining. Furthermore, immunohistochemical staining was used to measure the expression level of Beclin-1 and immunofluorescence labeling was used to visualize LC3 expression, and western blotting was used to determine the expression levels of autophagy proteins and key proteins in the PI3K signaling pathway. The apoptotic rate of OA chondrocytes was markedly elevated by 3-methyladenine and suppressed by rapamycin and icariin; autophagy genes were drastically downregulated in the 3-methyladenine group and upregulated in the rapamycin and icariin groups; and the PI3K/AKT/mTOR signaling pathway was activated by 3-methyladenine and inhibited by rapamycin and icariin. Notably, following treatment with rapamycin and icariin, the severe pathological state in OA cartilage tissues was substantially alleviated, and this was accompanied by activated autophagy and inhibited PI3K signaling in the cartilage tissues. Taken together, these findings indicate that icariin alleviates OA by regulating the autophagy of chondrocytes by mediating PI3K/AKT/mTOR signaling.

## Graphical abstract

**Figure:**
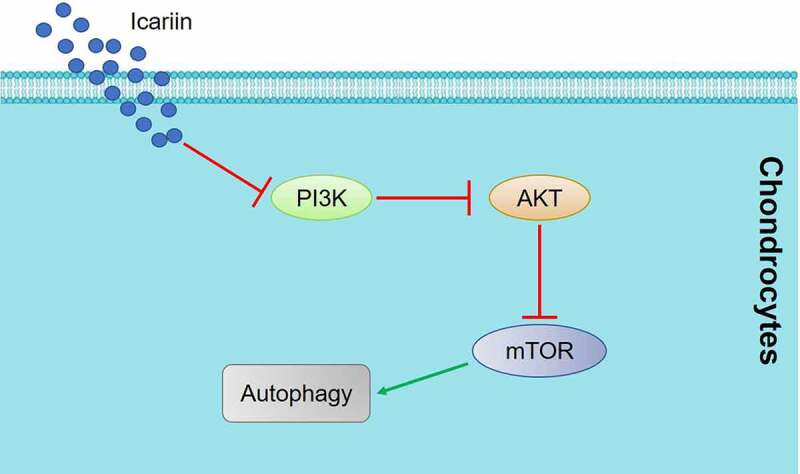

## 1. Introduction

Osteoarthritis (OA) is a common chronic degenerative disease that is mainly characterized by joint pain and swelling, as well as limited movements, which contribute to the decline in mobility and dysfunction in middle-aged and elderly people [1]. In China, the morbidity rate of primary OA in the population over 40 years old is ~46.3%, and this continues to increase annually [2]. Currently, the pathological mechanism underlying OA remains unclear; however, the function of chondrocyte autophagy in OA development and progression has recently attracted considerable research attention [3].

Autophagy is the process by which autologous substances in the cytoplasm are phagocytosed and then degraded in lysosomes, and autophagy is essential for normal cellular metabolism and the update of certain organelles [4]. Carames [5] reported that in patients with OA, the autophagy level of chondrocytes was considerably diminished, which was verified by the suppressed expression of the proteins ULK1, Beclin-1, and light chain 3 (LC3). Similar results were obtained with an experimental murine model of OA. Sasaki [6] reported that in chondrocytes exhibiting low levels of autophagy, interleukin-1β (IL-1β) could downregulate the expression of collagen II and aggrecan and upregulate the expression of matrix metalloproteinase (MMP) 13 and a disintegrin-like and metalloproteinase with thrombospondin motifs (ADAMTS), which contributed to OA progression. Furthermore, chondrocyte apoptosis and mitochondrial dysfunction tend to be induced by a reduced level of autophagy [7], and mitochondrial dysfunction is reported to be a key inducer of chondrocyte injury and cartilage degeneration [8]. Notably, when LC3 transgenic mice labeled with green fluorescence were used to dynamically monitor chondrocyte autophagy and OA cartilage injury, cartilage degeneration was found to be accompanied by a decreased level of autophagy [9]. These findings indicate a critical involvement of autophagy in the OA pathological process.

As an inhibitor of mTORC1, rapamycin has been reported to induce autophagy and has received increasing attention in the field of antiaging and OA treatment [10]. Sasaki [6] reported that rapamycin reversed the IL-1β-induced decrease in the expression of collagen II and aggrecan in human chondrocytes, and this was accompanied by suppressed expression of MMP13 and ADAMTS5. Moreover, rapamycin alleviated IL-1β-induced apoptosis and accumulation of reactive oxygen species, which prevented the chondrocyte damage triggered by mitochondrial dysfunction [7]. In this study, rapamycin was used as a positive control to induce chondrocyte autophagy, whereas 3-methyladenine, an inhibitor of autophagy, was used as a negative control to suppress autophagy.

Icariin – one of the main components of epimedium – is an 8-isopentene flavonoside that has been reported to produce broad pharmacological effects, such as improvement of cardiovascular function, promotion of hematopoietic function, protection against neuronal damage, anti-osteoporosis effects, and enhancement of sexual function [11]. Recently, the effects of icariin on autophagy activation have been widely reported [12,13], and, furthermore, the therapeutic effects of icariin on OA have been illustrated [14,15]. Mi [13] reported that icariin alleviates NF-κB signaling-mediated apoptosis in chondrocytes by activating autophagy and might serve as a promising compound for cartilage tissue engineering in OA treatment. Moreover, icariin was found to enhance chondrocyte vitality by promoting hypoxia-inducible factor-1α expression and anaerobic glycolysis, which again makes icariin a promising candidate for OA treatment [16]. Here, we suspected that icariin could exert anti-OA effects by regulating autophagy in chondrocytes. Thus, the protective effect of icariin against OA and the potential underlying mechanism were investigated to enhance our understanding of the therapeutic property of icariin in OA and to provide a fundamental basis for the clinical application of icariin in OA treatment.

## 2. Materials and methods

Ethics statement: We declare that all animal experiments involved in this study were authorized by the ethics committee of the Hospital of Traditional Chinese Medicine of Xiaoshan District and were performed according to the guidelines for the care and use of laboratory animals, as well as to the principles of laboratory animal care and protection.

### 2.1. Establishment of OA rat model

The rat OA model was established according to the instructions reported previously [17]. Sprague-Dawley rats were purchased from Beijing Vital River Laboratory Animal Technology Co., Ltd. After anesthetizing the animals by intraperitoneally injecting 45 mg/kg 2% pentobarbital sodium, a longitudinal incision was made on the inner skin of the right knee, and then the medial collateral ligaments were cut off and the articular cavity was opened. Subsequently, the medial meniscus was removed and the anterior cruciate ligaments were cut off, after which the incisions were sutured. An appropriate amount of penicillin was injected to prevent infection at 3 days post-surgery. The animals were driven out to run for consecutive 30 min daily starting from the fourth day post-surgery. The duration of the modeling was approximately two months. For the animals in the sham group, a longitudinal incision was made on the inner skin of the right knee, but no other surgical procedure was performed before suturing the incision.

### 2.2. Grouping of animals for experiments

The rats were divided into 6 groups (n = 6/group) and subject to the following treatments: Sham group: intraperitoneal injection of 10 mL/kg normal saline daily for 4 consecutive weeks; rapamycin group: intraperitoneal injection of 1 mg/kg rapamycin twice weekly for 4 consecutive weeks; 3-methyladenine group: intraperitoneal injection of 30 mg/kg 3-methyladenine twice weekly for 4 consecutive weeks; icariin groups: intraperitoneal injection of 20, 40, or 80 mg/kg/day icariin for 4 consecutive weeks.

### 2.3. Isolation and culture of chondrocytes

The OA rats were sacrificed by injecting an overdose of 2% pentobarbital sodium (200 mg/kg) and then the knee joint was isolated under sterile conditions. The cartilage tissues of the joint were separated under a microscope, and then 0.25% pancreatin was used to digest the tissues for 30 min. Next, the tissues were digested for another 4 h (at 37°C) with 0.15% collagenase II, after which the separated cells were filtered using a strainer (200 mesh). Lastly, the isolated chondrocytes were cultured in DMEM containing 10% FBS at 37°C and 5% CO_2_. The isolated chondrocytes were identified by performing immunofluorescence labeling with a collagen II antibody.

### 2.4. CCK-8 assay

The viability of treated chondrocytes was assessed using the CCK-8 assay. Briefly, cells were seeded in 96-well plates at a density of 5 × 10^4^ cells/well and incubated for 24 h. Subsequently, 30 μL of CCK-8 solution was added to each well, and after incubation at 37°C for 4 h, the absorbance at 450 nm was measured using a microplate reader (BECAM-CULTER, GA, USA); the absorbance value was used for calculating cell viability according to the manufacturer’s instructions [18,19].

### 2.5. Acridine orange-propidium iodide (AO-PI) assay

Chondrocyte apoptosis was evaluated using the AO-PI double fluorescence staining assay [20]. Before the experiment, 1 μL each of AO dye and PI dye (both at 1 mg/mL) were mixed in PBS buffer. After chondrocytes were exposed to various treatments, the medium was removed and the cells were washed with PBS buffer, and then 1 mL of PBS buffer containing the AO dye and PI dye was added and the cells were incubated for 5 min at room temperature in the dark. Lastly, images of the chondrocytes were captured using a fluorescence inverted microscope (Olympus, Tokyo, Japan).

### 2.6. Flow cytometry

Chondrocyte apoptosis was confirmed using flow cytometry [21]. Treated cells were digested and then transferred to a centrifuge tube and centrifuged, and after washing twice, the cells were resuspended in 10 μL of Annexin V solution and incubated for 30 min at 4°C. Next, ~300 μL of Annexin-FITC buffer was added and then 0.5 mL of the binding buffer was added, and after transferring the mixture to an ice-cold flow tube, 15 μL of PI solution was added. Lastly, the samples were loaded on a flow cytometer (BD, New York, USA) for apoptosis analysis.

### 2.7. Real-time PCR assay

Total RNA was isolated from treated chondrocytes by using TRIzol solution (Thermo Fisher Scientific, MA, USA), and 2 μg of the total RNA from different samples was used for cDNA synthesis by using a Transcriptor First-Strand cDNA Synthesis Kit (Promega, WI, USA). Subsequently, PCR was performed using SYBR Green Master Mix (Takara, Tokyo, Japan). GAPDH gene expression was measured for normalization, and the 2^−ΔΔt^ method was used to calculate the relative expression level of target genes [22].

### 2.8. Western blotting assay

RIPA Lysis Buffer (Thermo Fisher Scientific) was used for total protein extraction from chondrocytes, and the extracted proteins were separated using SDS-PAGE and transferred to PVDF membranes, which were incubated in 5% milk for 2 h at room temperature to block nonspecific binding. After washing thrice with PBS buffer, the membranes were incubated (overnight at 4°C) with primary antibodies against autophagy-related gene (ATG) 7, LC3-II/LC3-I, PI3K, p-AKT1, AKT1, p-mTOR, mTOR, p70S6K, p-p70S6K, or GAPDH; all primary antibodies were from Cell Signaling Technology (CST; Boston, MA, USA) and were used at 1:1000 dilution. Next, the membranes were incubated with anti-rabbit IgG antibody (1:1000, CST) for 2 h at room temperature, and then the immunoreactive proteins on the membranes were detected using an imaging system (Bio-Rad, Hercules, CA, USA) [23].

### 2.9. Micro-CT scanning of knee joint

Excessive soft tissues around the knee joint were removed and then the knee joint was scanned using a uCT80 Micro-CT instrument (SCANCO, Brüttisellen, Switzerland) [24]. Distinguishability was set at 18 μm. The images were scanned layer by layer, and then 3D reconstruction was performed.

### 2.10. Safranin O staining

Samples were stained with Weigert’s iron hematoxylin, washed thrice with water, and then sequentially incubated in 0.2% fast green solution (Sangon, Shanghai, China) for 1 min, 1% ethylic acid solution for 30 s, and 0.1% safranin O solution (Sangon) for 15 min. Lastly, the samples were dehydrated, made transparent, and mounted with neutral balsam. The normal cartilage appeared red, and the background appeared green [25]. The pathological score was evaluated using the Osteoarthritis Research Society International (OARSI) scoring system described previously [26].

### 2.11. Immunohistochemical assay

Cartilage tissues were isolated and fixed in formalin, decalcified in formic acid, embedded in paraffin, and sectioned for immunohistochemical analysis. Briefly, sections were incubated with an antibody against Beclin-1 (CST) and then with anti-rabbit secondary antibody (CST), and the staining of sections was visualized using 3,3-diaminobenzidine chromogen (Vector Laboratories, CA, USA). Negative controls were stained with irrelevant isotype-matched antibodies (CST). Images were captured using a fluorescence inverted microscope (Olympus) [27].

### 2.12. Immunofluorescence assay

Briefly, sections were deparaffinized with xylene, washed twice with ethanol to remove the remaining xylene, and then sequentially dehydrated in 95%, 80%, and 70% ethanol solutions. The sections were next incubated with EDTA for 20 min, washed thrice with PBS buffer, blocked with 3% goat serum for 1 h at room temperature, and then incubated with an LC3-II antibody at 4°C overnight. After washing with PBS buffer thrice, FITC-labeled anti-rat secondary antibody was added and incubated at 37°C for 30 min. Lastly, the sections were sealed with neutral resins and examined under a laser-scanning confocal microscope (Olympus) [25].

### 2.13. Transfection

To establish PI3K-overexpressing chondrocytes, we generated a pcDNA3.1-PI3K recombined vector by inserting the PI3K sequence into the blank pcDNA3.1 vector; the blank vector served as the negative control (pcDNA3.1-NC). The vectors were transfected into chondrocytes by using Lipofectamine 3000 reagent (Thermo Fisher Scientific), and this was followed by incubation for 48 h. The transfection efficacy was evaluated through western blotting.

### 2.14. Statistical analysis

Data are presented as means ± standard deviation (SD). One-way analysis of variance and Student-Newman-Keuls multiple-comparison tests were used to analyze the significance of differences between the control and experimental groups; p < 0.05 was considered significant.

## 3. Results

We suspected that the protective effects exerted by icariin on OA might be related to the activation of autophagy in chondrocytes. This study was conducted to explore the potential therapeutic effect of icariin in OA rats and the underlying mechanism. We established the rat OA model and then in OA chondrocytes extracted from these animals, we investigated (1) the protective effect of icariin, (2) the regulatory effect of icariin on autophagy, and (3) the impact of icariin on PI3K/AKT/mTOR signaling. Subsequently, we examined the therapeutic effect of icariin in OA rats by evaluating the pathological state of cartilage tissues in conjunction with a measurement of autophagy and PI3K/AKT/mTOR signaling in the tissues. Lastly, we verified the involvement of PI3K/AKT/mTOR signaling in the mechanism of action of icariin by generating PI3K-overexpressing OA chondrocytes and examining the icariin effect in these cells.

### 3.1. Identification of isolated chondrocytes and determination of optimal drug concentrations

**Figure 1.**
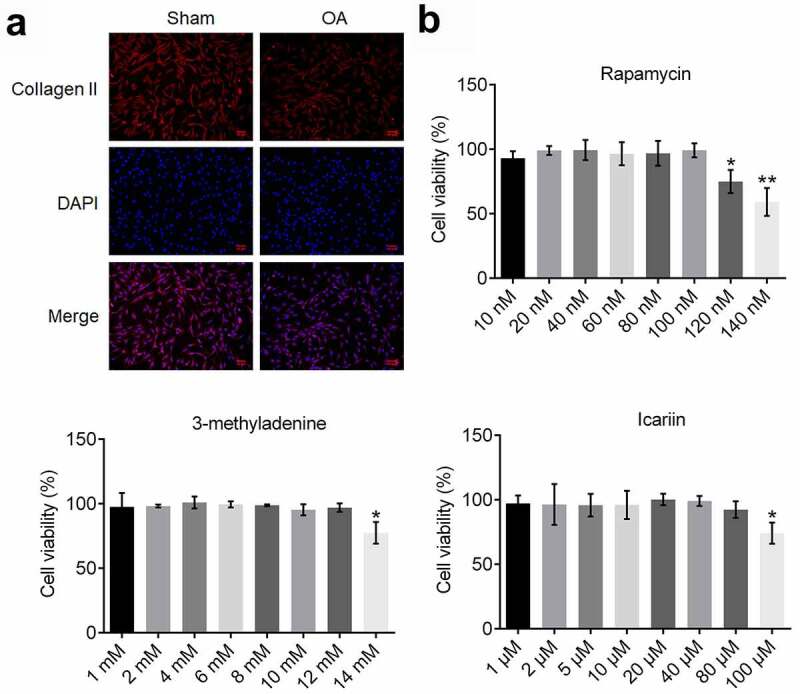
Identification of isolated chondrocytes and determination of optimal concentrations of drugs. (a). Collagen II expression was detected using an immunofluorescence assay. The expression level of collagen II was decreased in chondrocytes isolated from OA rats. (b). Viability of treated chondrocytes was measured using the CCK-8 assay. The highest concentration at which rapamycin, 3-methyladenine, and icariin were incubated with chondrocytes was 100 nM, 12 mM, and 80 μM, respectively (*p < 0.05, **p < 0.01). Data are presented as means ± SD (n = 6)

To identify the isolated chondrocytes, we performed an immunofluorescence assay by using a collagen II antibody (Figure 1(a)); red fluorescence, indicating staining of collagen II, was observed in cells isolated from both sham- and OA-group rats. Moreover, the intensity of red fluorescence was markedly lower in chondrocytes isolated from the model rats than in the cells from the sham group; this indicated successful establishment of the OA model in rats because collagen II degradation in chondrocytes is reported to represent a key characteristic of OA [28].

To determine the optimal concentration of rapamycin, 3-methyladenine, and icariin for use in assays, chondrocytes were incubated with different concentrations of these reagents and then cell viability was evaluated (Figure 1(b)): Cell viability was not markedly altered as the rapamycin concentration was increased from 10 to 100 nM, but was significantly decreased as the concentration was increased to 120 and 140 nM (*p < 0.05 and **p < 0.01 vs. 10 nM). Furthermore, cell viability was drastically decreased as the concentration of 3-methyladenine was increased from 12 to 14 mM (*p < 0.05 vs. 1 mM) and that of icariin was increased from 80 to 100 μM (*p < 0.05 vs. 1 μM). Therefore, 100 nM rapamycin, 12 mM 3-methyladenine, and 20, 40, and 80 μM icariin were used in this study.

### 3.2. Icariin alleviated apoptosis in OA chondrocytes

Considerable chondrocyte apoptosis is observed during the development of OA [29]. Thus, we first examined the effect of icariin on the apoptosis of OA chondrocytes. For the in vitro experiments, we sorted OA chondrocytes into six groups, with the cells in these groups being incubated with blank medium (control), 100 nM rapamycin (rapamycin), 12 mM 3-methyladenine (3-methyladenine), or 20, 40, or 80 μM icariin. AO-PI staining and flow cytometry were performed on the treated chondrocytes to evaluate apoptosis.

**Figure 2.**
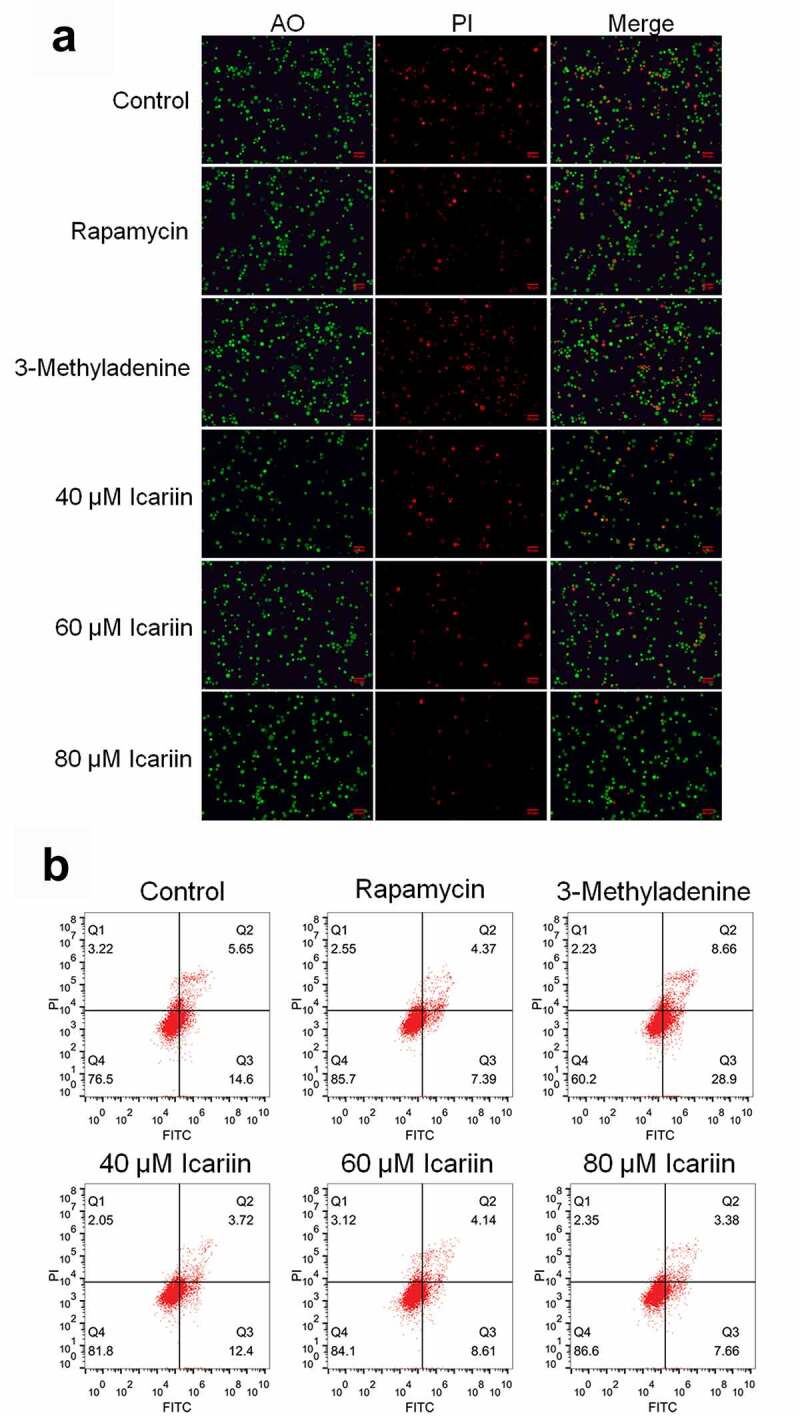
Apoptosis in OA chondrocytes was significantly alleviated by icariin. (a). AO-PI staining was used to evaluate the apoptotic state of treated chondrocytes. Fluorescence intensity was decreased in the rapamycin and icariin groups and increased in the 3-methyladenine group. (b). Flow cytometry was used to determine the apoptotic rate of treated chondrocytes. Apoptotic rate was decreased in the rapamycin and icariin groups and increased in the 3-methyladenine group

Relative to the control level (in untreated OA chondrocytes), the proportion of apoptotic chondrocytes was increased among 3-methyladenine-treated OA chondrocytes and decreased among rapamycin-treated OA chondrocytes, and with an increase in icariin concentration, the proportion of apoptotic chondrocytes was decreased in a dose-dependent manner (Figure 2(a)). Furthermore, relative to the control, the apoptotic rate of OA chondrocytes (Figure 2(b)) showed an increase from 20.25% to 37.56% after 3-methyladenine treatment and a decrease to 11.76% and 16.12%, 12.75%, and 11.04% after treatment with rapamycin and 20, 40, and 80 μM icariin, respectively. These data indicated that apoptosis in OA chondrocytes was potently alleviated by icariin, which agrees with the results described previously [13,30].

### 3.3. Icariin induced autophagy activation in OA chondrocytes

**Figure 3.**
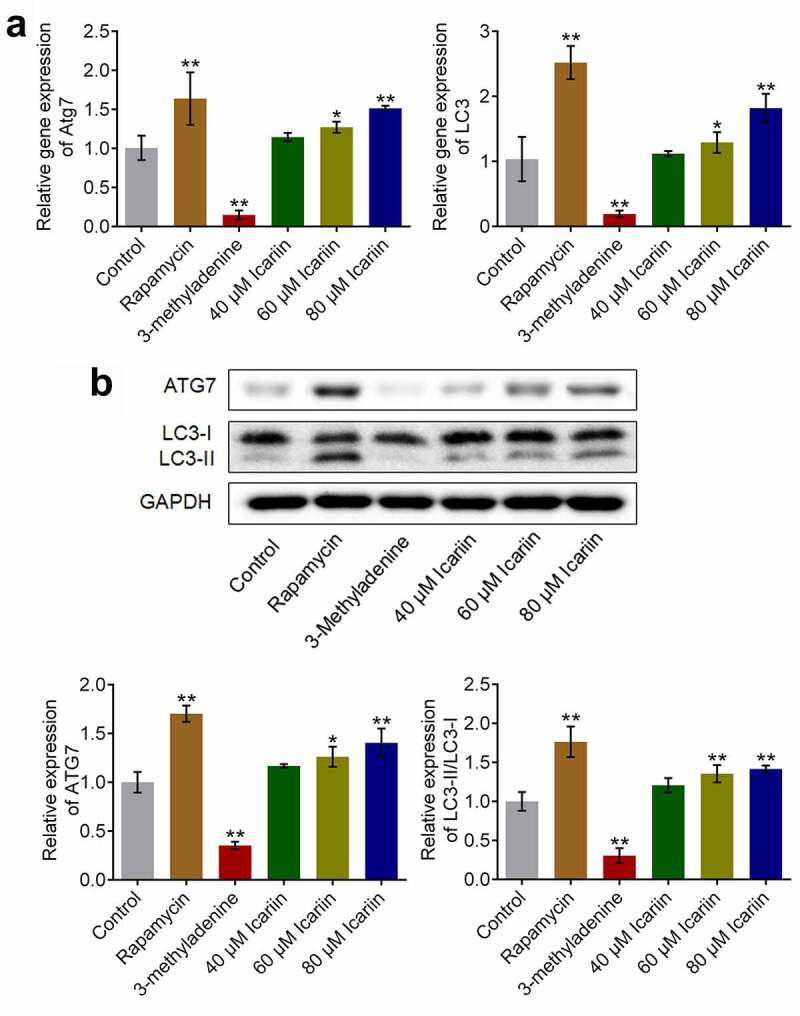
Autophagy in OA chondrocytes was activated by icariin. (a). Gene expression of *Atg7* and *LC3* was detected using qRT-PCR. *Atg7* and *LC3* expression was downregulated in the 3-methyladenine group and upregulated in the rapamycin and icariin groups (*p < 0.05 and **p < 0.01 vs. Control). (b). ATG7 and LC3-II/LC3-I protein levels were measured using western blotting. ATG7 and LC3-II/LC3-I levels were downregulated in the 3-methyladenine group and upregulated in the rapamycin and icariin groups (*p < 0.05 and **p < 0.01 vs. Control). Data are presented as means ± SD (n = 6)

Autophagy is reported to be critical for the self-repair of cells [31]. To evaluate how icariin affects chondrocyte autophagy, we determined the gene and protein expression levels of autophagy-related molecules. Relative to the control, the gene expression of *Atg7* and *LC3* was significantly suppressed in the 3-methyladenine group and elevated in the rapamycin group and the 40 and 80 μM icariin groups (*p < 0.05 and **p < 0.01 vs. control) (Figure 3(a)). Moreover, ATG7 and LC3-II protein expression was drastically downregulated in the 3-methyladenine group and upregulated in the rapamycin group and the 40 and 80 μM icariin groups (*p < 0.05 and **p < 0.01 vs. control) (Figure 3(b)). These results indicated that icariin strongly activated autophagy in OA chondrocytes. Facilitating effects of icariin on autophagy in chondrocytes were also reported by Mi [13] in 2018.

### 3.4. Icariin suppressed PI3K/AKT/mTOR signaling in OA chondrocytes

**Figure 4.**
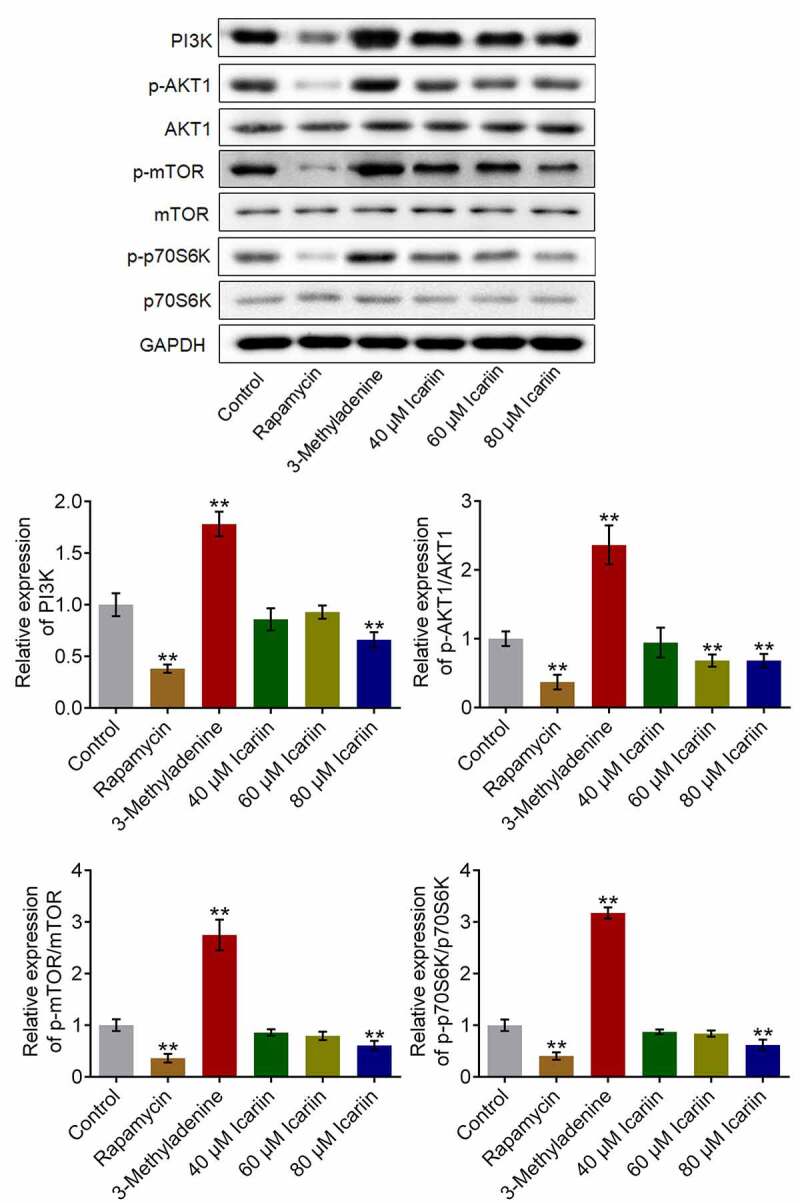
PI3K/AKT/mTOR signaling was drastically suppressed by icariin. Western blotting was used to determine the protein levels of PI3K, p-AKT1, AKT1, p-mTOR, mTOR, p70S6K, and p-p70S6K in treated chondrocytes. PI3K, p-AKT1/AKT1, p-mTOR/mTOR, and p70S6K/p-p70S6K levels were significantly decreased in the rapamycin and icariin groups and increased in the 3-methyladenine group (**p < 0.01 vs. Control). Data are presented as means ± SD (n = 6)

PI3K/AKT/mTOR signaling represents a critical signaling pathway that regulates the progression of autophagy [32]. Thus, to investigate the potential mechanism of action of icariin, we examined the effect of icariin on the PI3K/AKT/mTOR signaling pathway (Figure 4): Relative to the control, the levels of PI3K, p-AKT1, p-mTOR, and p-p70S6K were significantly increased in chondrocytes treated with 3-methyladenine and decreased in chondrocytes treated with rapamycin or 80 μM icariin (**p < 0.01 vs. control), which indicated that PI3K/AKT/mTOR signaling was potently suppressed by the icariin treatment. As an antitumor agent, icariin has also been reported to exert an inhibitory effect on human cervical cancer cells [33].

### 3.5. Icariin ameliorated pathological symptoms in OA rats

**Figure 5.**
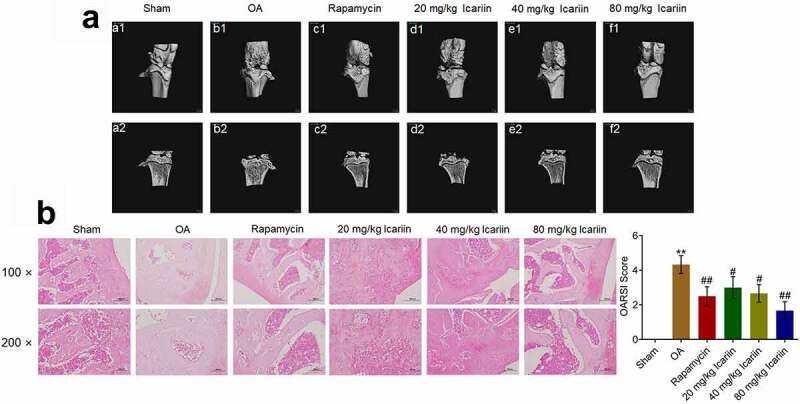
Icariin markedly ameliorated the pathological state of cartilage tissues in OA rats. (a). Pathological changes of articular cartilage were visualized using micro-CT scanning. The rough surface, focal plane cracks, exposed subchondral bone, oval osteophytes in articular cartilage and thin cartilage layer, disordered arrangement of bone trabeculae, and cystic subchondral bone on articular head were significantly alleviated in the rapamycin and icariin groups. (b). Pathological state of cartilage tissues was evaluated using safranin O staining. Cartilage degeneration and loss of proteoglycan, surface layer, and fiber layer in OA rats were notably ameliorated in the rapamycin and icariin groups

We analyzed the potential therapeutic effect of icariin on OA by exposing OA rats to different treatments (described in Section 2). Our results (Figure 5(a)) showed that in the sham group, a smooth surface with no osteophyte was present on the articular cartilage (a1), and properly aligned thick bone trabeculae were detected on the articular head (b1); by contrast, in the OA group, a rough surface, focal plane cracks, exposed subchondral bone, and oval osteophytes were observed on the articular cartilage (b1), and a thin cartilage layer, disordered arrangement of bone trabeculae, and cystic subchondral bone were observed on the articular head (b2). However, treatment with rapamycin significantly alleviated the pathological changes in the articular cartilage and head, and, notably, with an increase in icariin concentration, the pathological changes in the articular cartilage and head were ameliorated in a dose-dependent manner. The alleviation effect of rapamycin was also reported by Bao [34].

We further assessed the degree of tissue lesion by performing safranin O staining (Figure 5(b)). In the sham group, the surface layer and fiber layer were aligned, whereas in the OA group, marked cartilage degeneration and loss of proteoglycan, surface layer, and fiber layer were observed. Rapamycin administration markedly ameliorated the cartilage degeneration and fiber-layer loss, and this amelioration was also induced by icariin treatment in a dose-dependent manner. Relative to the sham group, the OA group featured a significantly elevated OARSI score, which was substantially diminished in the rapamycin and icariin groups (**p < 0.01 vs. sham, #p < 0.05 and ##p < 0.01 vs. OA). These data indicated that the pathological symptoms in OA animals were drastically alleviated by icariin.

### 3.6. Icariin activated autophagy in cartilage tissues

**Figure 6.**
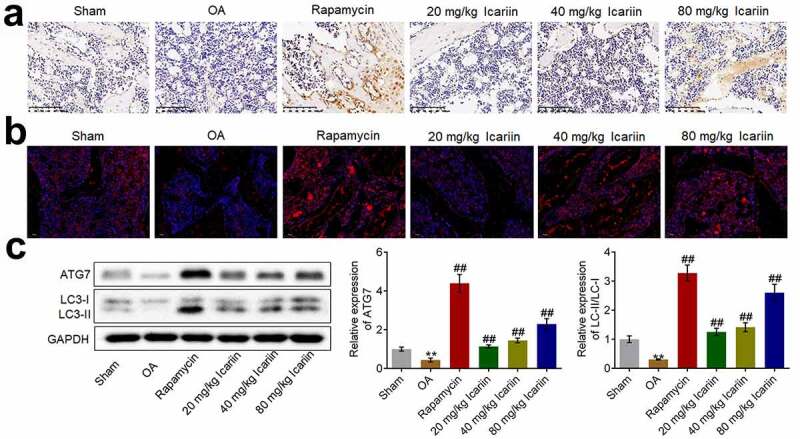
Icariin activated autophagy in OA chondrocytes. (a). Beclin-1 expression level was measured using an immunohistochemical assay. Beclin-1 was upregulated in the rapamycin and icariin groups. (b). LC3 expression was visualized using an immunofluorescence assay. LC3 was upregulated in the rapamycin and icariin groups. (c). Western blotting was used to assess the expression of ATG7, LC3-I, and LC3-II in cartilage tissues. ATG7 and LC3-II/LC3-I levels were elevated in the rapamycin and icariin groups (**p < 0.01 vs. Sham, ##p < 0.01 vs. OA). Data are presented as means ± SD (n = 6)

Considering the impact of icariin on autophagy in OA chondrocytes, we investigated the state of autophagy in cartilage tissues by determining the expression levels of Beclin-1, LC3, and ATG7. Beclin-1 expression was significantly lower in the OA group than in the sham group, whereas the expression was markedly elevated after rapamycin treatment and increased in a dose-dependent manner after icariin treatment (Figure 6(a)). Furthermore, the results of immunofluorescence and western blotting assays (Figure 6(b,c)) showed that ATG7 and LC3-II/LC3-I levels were markedly lower in the OA group than in the sham group, but the levels were significantly upregulated after rapamycin treatment and increased in a dose-dependent manner after icariin treatment (**p < 0.01 vs. sham, ##p < 0.01 vs. OA). The autophagy-inducing effect of rapamycin in OA cartilage tissues has also been reported by Bao [34].

### 3.7. Icariin suppressed PI3K/AKT/mTOR signaling in OA cartilage tissues

**Figure 7.**
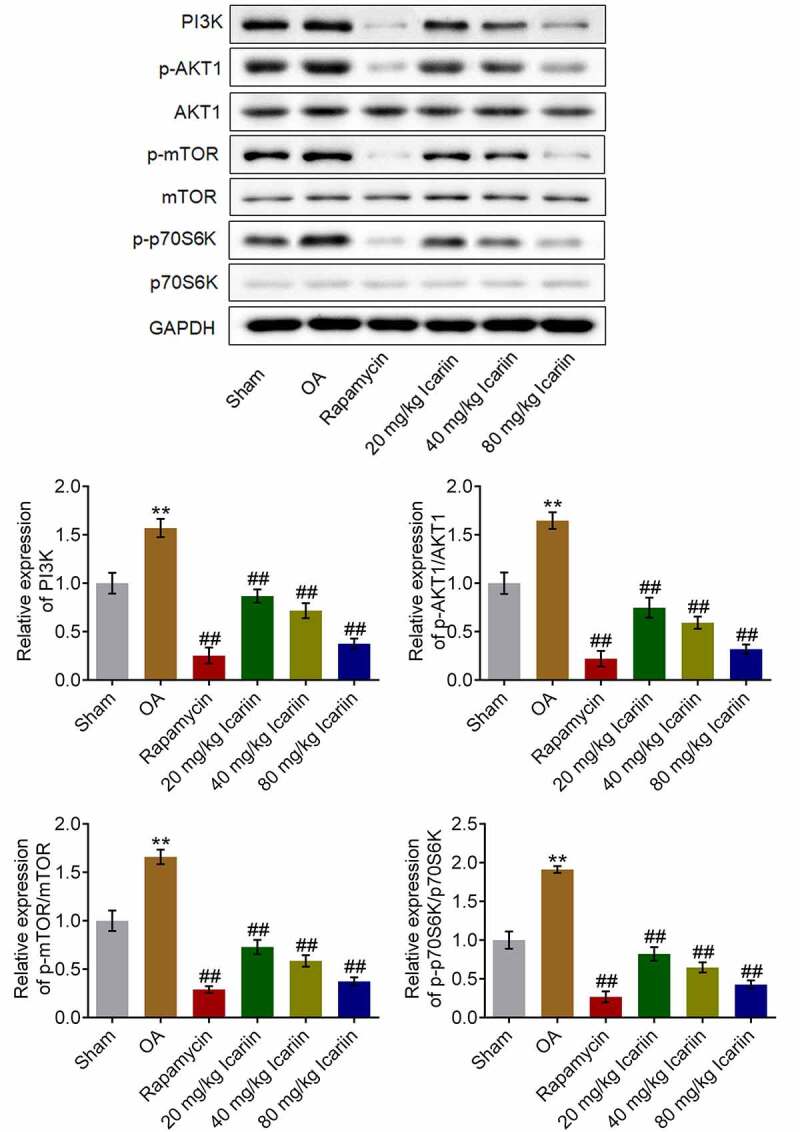
Icariin potently suppressed PI3K/AKT/mTOR signaling in cartilage tissues. Western blotting was used to measure the protein levels of PI3K, p-AKT1, AKT1, p-mTOR, mTOR, p70S6K, and p-p70S6K in cartilage tissues. PI3K, p-AKT1/AKT1, p-mTOR/mTOR, and p70S6K/p-p70S6K levels were significantly suppressed in the rapamycin and icariin groups (**p < 0.01 vs. Sham, ##p < 0.01 vs. OA). Data are presented as means ± SD (n = 6)

We further investigated the impact of icariin on the PI3K/AKT/mTOR signaling pathway in cartilage tissues. The protein levels of PI3K, p-AKT1, p-mTOR, and p-p70S6K were significantly higher in the OA group than in the sham group, but these levels were drastically decreased after rapamycin treatment and decreased in a dose-dependent manner after icariin administration (**p < 0.01 vs. sham, ##p < 0.01 vs. OA) (Figure 7). These results indicated that the PI3K/AKT/mTOR signaling pathway in OA cartilage tissues was strongly suppressed by icariin treatment.

### 3.8. Protective effects and autophagy-facilitating effects of icariin on OA chondrocytes were abolished by PI3K overexpression

**Figure 8.**
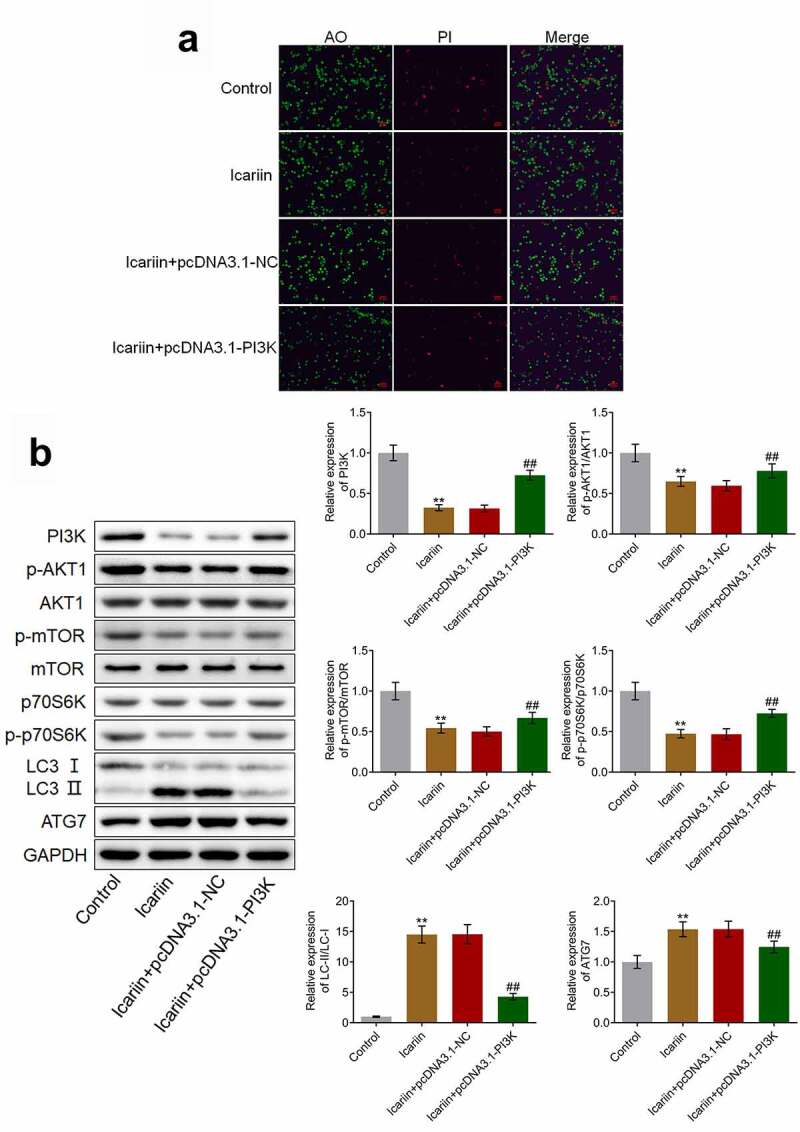
PI3K overexpression abolished the protective effect and autophagy-promoting effect of icariin on OA chondrocytes. (a). AO-PI staining was used to evaluate the apoptotic state of treated chondrocytes. The proportion of apoptotic chondrocytes was decreased following icariin treatment, but the effect was reversed by the overexpression of PI3K. (b). Western blotting was used to determine the protein levels of PI3K, p-AKT1, AKT1, p-mTOR, mTOR, p70S6K, p-p70S6K, LC3, and ATG7. PI3K, p-AKT1, p-mTOR, and p-p70S6K levels were significantly decreased and LC3-II/LC3-I and ATG7 levels were drastically increased in the icariin group, but these effects were reversed by PI3K overexpression (**p < 0.01 vs. Control, ##p < 0.01 vs. icariin + pcDNA3.1-NC)

To verify that icariin exerted a protective effect on chondrocytes by regulating PI3K/AKT/mTOR signaling, we established PI3K-overexpressing OA chondrocytes. Our results (Figure 8(a)) demonstrated that relative to the control (untreated OA chondrocytes), the OA chondrocytes treated with 40 μM icariin showed a significant decrease in the proportion of apoptotic chondrocytes; however, relative to the icariin + pcDNA3.1-NC group, the icariin + pcDNA3.1-PI3K group showed a significant increase in the proportion of apoptotic chondrocytes. Furthermore, as compared with the icariin + pcDNA3.1-NC group, the icariin + pcDNA3.1-PI3K group showed a significant increase in the levels of PI3K, p-AKT1, p-mTOR, and p-p70S6K and a drastic decrease in the levels of LC3-II/LC3-I and ATG7 (**p < 0.01 vs. control, ##p < 0.01 vs. icariin + pcDNA3.1-NC) (Figure 8(b)). These results indicated that the inhibitory effect of icariin on the PI3K/AKT/mTOR signaling pathway and the activation effect of icariin on autophagy were abolished by the overexpression of PI3K.

## 4. Discussion

Autophagy regulates the mechanism of ‘self-digestion and balance’ in normal articular cartilage, which maintains cellular homeostasis by removing misfolded proteins, damaged organelles, and macromolecules through lysosome-dependent autophagy, synthesizing new proteins, suppressing the processing of apoptosis, and providing essential raw materials for cellular reconstruction, regeneration, and repair [35]. The main mediators involved in the regulation of autophagy include ATGs, Beclin-1, and LC3. Disruption of the Beclin-1-Bcl-2 complex is regarded as a critical mechanism for the activation of autophagy in mammals [36]. LC3 mainly exists in two forms, LC3-I and LC3-II, with LC3-I localizing in the cytoplasm as a hydrophilic molecule and LC3-II functioning in the formation of the autophagosome, which is derived from the esterification of LC3-I through a ubiquitin-like system; LC3-II ultimately combines with autophagic vacuoles to induce autophagy activation. Therefore, LC3-II is widely considered to be a key biomarker of autophagy [37].

In the pathological process of OA, the activity of chondrocyte autophagy cannot be maintained at normal levels, and this contributes to the decline in cell viability and the initiation of apoptosis. Cartilage degeneration can be aggravated by excessive production of damaged organelles and unnecessary macromolecules, but these are typically removed by autophagy, and, consequently, the survival rate of chondrocytes is elevated and the progression of OA is suppressed [38]. Here, we established an OA animal model and found that autophagy activity was decreased in the articular cartilage tissues of the OA rats; this was verified by the decreased expression levels of ATG7, LC3-II/LC-I, and Beclin-1.

In this study, we used rapamycin, an autophagy activator, and 3-methyladenine, an autophagy inhibitor, to illustrate the function of autophagy in OA progression. In chondrocytes isolated from OA rats, autophagy was strongly activated by rapamycin treatment, and this was accompanied by a decreased apoptotic rate; by contrast, autophagy was drastically suppressed by 3-methyladenine treatment, and this was accompanied by an increased apoptotic rate. These data agree with those reported previously [39,40].

We found that icariin activated autophagy in chondrocytes and decreased their apoptotic rate in a dose-dependent manner, indicating that icariin might protect chondrocytes by activating autophagy. Verification experiments on OA rats confirmed that rapamycin and icariin produced a therapeutic effect against OA and that this was accompanied by the activation of autophagy in cartilage tissues. In future work, we will conduct more comprehensive evaluations to confirm the therapeutic activity of icariin toward OA, including X-ray callus examination and inflammation detection.

The PI3K/AKT/mTOR signaling pathway is reported to be involved in gene transcription, protein translation, and ribosome biogenesis, and this plays a crucial role in the regulation of autophagy [41]. PI3K activated by the growth factor receptor signaling pathway induces the production of phosphatidylinositol 3,4,5-trisphosphate (PIP3) to activate PDK1, which further triggers AKT phosphorylation; consequently, mTORC1 is activated and contributes to the suppression of autophagy [42]. Guowang reported that curcumin alleviated the apoptosis of chondrocytes by elevating the level of autophagy by inhibiting the PI3K/AKT/mTOR signaling pathway [43].

In this study, the PI3K/AKT/mTOR signaling pathway was found to be markedly suppressed in OA chondrocytes by activators of autophagy (rapamycin and icariin) and drastically activated by an inhibitor of autophagy (3-methyladenine); this indicated that icariin might regulate the processing of autophagy by inhibiting the PI3K/AKT/mTOR signaling pathway. Subsequently, animal experiments confirmed the involvement of PI3K/AKT/mTOR signaling in the regulatory effect of icariin on autophagy in cartilage tissues. The results of our in vitro verification experiments showed that both the protective effect and the autophagy-activation effect of icariin on OA chondrocytes were abolished following PI3K overexpression, which indicates that the biofunction of icariin is mediated by the inhibition of the PI3K/AKT/mTOR signaling pathway. Collectively, the findings of this study indicate that icariin alleviates OA symptoms by regulating the autophagy of chondrocytes by mediating PI3K/AKT/mTOR signaling.

## Data Availability

Part of the data can be shared if it is requested from the editor.

## Highlights

- Icariin alleviates osteoarthritis.
- Icariin activates autophagy in chondrocytes.
- Icariin mediates PI3K/AKT/mTOR signaling.
